# Selected Data Mining Tools for Data Analysis in Distributed Environment

**DOI:** 10.3390/e24101401

**Published:** 2022-10-01

**Authors:** Mikhail Moshkov, Beata Zielosko, Evans Teiko Tetteh

**Affiliations:** 1 Computer, Electrical and Mathematical Sciences and Engineering Division and Computational Bioscience Research Center, King Abdullah University of Science and Technology (KAUST), Thuwal 23955-6900, Saudi Arabia; 2 Institute of Computer Science, Faculty of Science and Technology, University of Silesia in Katowice, Bȩdzińska 39, 41-200 Sosnowiec, Poland; 3 Doctoral School, University of Silesia in Katowice, Bankowa 14, 40-007 Katowice, Poland

**Keywords:** distributed data, decision tables, information systems, decision trees, decision rules, tests, reducts, association rules

## Abstract

In this paper, we deal with distributed data represented either as a finite set T of decision tables with equal sets of attributes or a finite set I of information systems with equal sets of attributes. In the former case, we discuss a way to the study decision trees common to all tables from the set T: building a decision table in which the set of decision trees coincides with the set of decision trees common to all tables from T. We show when we can build such a decision table and how to build it in a polynomial time. If we have such a table, we can apply various decision tree learning algorithms to it. We extend the considered approach to the study of test (reducts) and decision rules common to all tables from T. In the latter case, we discuss a way to study the association rules common to all information systems from the set I: building a joint information system for which the set of true association rules that are realizable for a given row ρ and have a given attribute *a* on the right-hand side coincides with the set of association rules that are true for all information systems from I, have the attribute *a* on the right-hand side, and are realizable for the row ρ. We then show how to build a joint information system in a polynomial time. When we build such an information system, we can apply various association rule learning algorithms to it.

## 1. Introduction

Along with technological development, we are dealing with an increasing amount of data that must be processed and stored. The way they are processed depends on many factors, including the purpose of use and the type of data. One of the main goals is to extract knowledge from data, for example, by discovering patterns and relationships hidden in the data. Such knowledge can be presented by a set of decision rules, decision trees, or association rules. When a selection of features is required in order to find the most important and relevant ones, a test (reduct) is used. It is a (minimal) set of attributes that provides the same classification of objects as the whole input set of features.

An important element that influences the result of the chosen approach to extracting knowledge from data is their preparation. Pre-processing includes various algorithms, depending on the needs. These can be, for example, the imputation of missing attribute values, data normalization, or discretization. The type of method used depends on the goal and affects the subsequent stages of the data mining process. This phase is particularly difficult when we are dealing with distributed data that come from various data sources and appear in a different format, depending on the data owner [1].

One popular form of data representation is the tabular form, presented either as a decision table or as an information system. In the case of a distributed environment, such data can be represented as a finite set of decision tables with the same decision attribute [2,3]. Generally, these decision tables can have different sets of conditional attributes. However, the consideration of the sets of decision tables with equal sets of attributes is of particular interest. Data can also be represented by information systems [4,5]. As for the case of decision tables, considering the sets of information systems with equal sets of attributes is of most interest to us. This paper consists of the two parts. In the first one, we deal with dispersed data represented by a finite set of decision tables with equal sets of attributes. In the second part, we deal with dispersed data represented by a finite set of information systems with equal sets of attributes.

In the first part of the paper, we assume that we have a finite set $T=\{T_{1},\ldots ,T_{k}\}$ of decision tables with equal sets of attributes. Our aim is to create tools for the work with decision trees, rules, and tests (reducts) [4,5,6] that are common to all decision tables from T.

There are different algorithms for the construction and optimization of decision trees for single decision tables [7,8,9,10]. To apply these algorithms to the set of decision tables T, we need to build a single decision table (called a joint decision table for T) such that the set of decision trees for this table is equal to the set of common decision trees for all decision tables from T. The situation is the same for decision rules and tests (reducts). In this paper, we show when we can build joint decision tables and how to build them in a polynomial time.

Note that in the case of dispersed decision tables with different sets of conditional attributes, instead of considering a joint decision table, we should study its lower and upper approximations, which leads to the investigation of NP-hard problems [2].

In the second part of the paper, we assume that we have a finite set $I=\{I_{1},\ldots ,I_{k}\}$ of information systems, in which columns are labeled with the same attributes $a_{1},\ldots ,a_{n}$. We fix a row ρ from one of the information systems from I and an attribute $a_{j}\in \left\{a_{1},\ldots ,a_{n}\right\}$, and we consider the set $Arules(I,\rho ,a_{j})$ of association rules of the form $\left(a_{i_{1}}=\sigma_{1}\right)\wedge \cdots \wedge \left(a_{i_{m}}=\sigma_{m}\right)\to \left(a_{j}=\sigma \right)$ that are true for each information system from I and are realizable for the row ρ (i.e., such rule covers the row ρ). Our aim is to create tools for the work with association rules from this set.

There are different algorithms for the construction and optimization of association rules for single information systems [11,12,13,14,15,16]. To apply these algorithms to the set of information systems I, we need to build an information system *J* (called a joint information system for I,ρ, and $a_{j}$) such that $Arules\left(\left\{J\right\},\rho ,a_{j}\right)=Arules\left(I,\rho ,a_{j}\right)$. In this paper, we show how to build joint information systems in a polynomial time.

The main contribution of this work is a proposed new methodology for working with distributed data, presented as a set of decision tables or a set of information systems. It is an interesting direction of research, especially in the areas of distributed data mining, data processing, and knowledge extraction from dispersed data sources. The proposed approach is different from the approaches described in the framework of distributed data mining (Section 2.1). Our methodology is based on the transformation of distributed data sources into the so-called joint tabular form of data, presented as a joint decision table or as a joint information system. An important element is that the obtained decision table or information system allows for the induction of decision rules, decision trees, reducts, or association rules common to the distributed data. Moreover, existing algorithms for their induction can be used.

The present paper is an extended version of two conference papers [17,18].

The rest of the paper is organized as follows. Section 2 presents some background information related to distributed data, decision trees and rules, tests, and reducts as well as association rules. In Section 3, we study distributed data represented as a finite set of decision tables, and in Section 4, we study distributed data represented as a finite set of information systems. Section 5 contains brief conclusions.

## 2. Background Information

In this section some basic information related to distributed data, decision trees and rules, tests, and reducts as well as association rules is presented.

### 2.1. Distributed Data

Technological development means that we are dealing with an increasing amount of data that can be heterogeneous, taking into account their format and location.

One of the popular solutions for processing and storing decentralized data are data warehouses [19,20]. They are used to store huge data sets. By using appropriate analytical tools that allow for the employment of data mining algorithms, it is possible to mine knowledge from data by analyzing trends, anomalies, or searching patterns. On this basis, business decisions are made regarding, for example, sales planning or marketing campaigns. In addition, data warehouses have ETL (Extraction, Trasformation, Loading) tools, which are designed to properly prepare data from heterogeneous sources and various locations.

Along with technological development and the necessity to process large amounts of distributed data, the field referred to as distributed data mining has been developing in recent years [21,22]. In this framework, different algorithms and approaches have been developed and proposed for classification, association mining, clustering, and other data mining tasks [23,24].

In this paper, a new methodology for working with distributed data is proposed. It is based on the idea of constructing one tabular form of data representation, i.e, a decision table or an information system for distributed sources, and then applying known algorithms for the induction of data mining tools, i.e., association and decision rules, decision trees, and reducts.

It should also be taken into account that distributed data mining techniques are more complex in comparison to centralized ones. The main issues which should be considered are: (i) heterogeneous data, i.e., local data sources can provide data with different formats and attributes with different domains; (ii) data fragmentation, i.e., local sources can be viewed as a horizontal or vertical fragmentation of the global data table, and therefore based on them, only part of the knowledge can be induced; (iii) data replication, i.e., replication provides better data availability, but on the other hand, it can make it difficult to ensure the consistency of distributed data; (iv) cost of communication in a distributed environment plays an important role; (v) security, privacy, and autonomy of local sources; (vi) integration results, i.e., discovered global interesting patterns and associations should be collected from local sources, and their utility should be verified globally.

Distributed data mining aims to analyze and process distributed data while taking into account resource constraints [25]. This task can be realized in the framework of a meta-learning, multi-agent system, or based on grid. The multi-agent data mining environment inherits properties of agents as interoperability and performance aspects. Interoperability concerns working collaboratively with other agents in the entire system. Performance measures can be improved or impaired by the data distribution at the local level. The meta-learning system constitutes a learning method at the local level. Learning at the meta level is based on accumulating experience on the performance of multiple applications of a learning system. Data mining based on grid aims to create a distributed computing environment in order to enable local data sources to use computing resources on demand.

### 2.2. Data Mining Tools

Data mining is a complex process that allows for the performance of analyses and the acquisition of knowledge from data by using different methods, depending on the aim and kind of data. Among data mining tools, decision rules, decision trees, reducts, and association rules can be used. They can be considered as algorithms for solving different problems and also as classifiers used in the area of machine learning [26]. A short description can be found in the sections below.

#### 2.2.1. Decision Rules

Decision rules are popular and an often used form of knowledge representation. In general, decision rules can be presented in the following form:

(1) $$IF\;condition_{1}\wedge \ldots \wedge condition_{k}\;THEN\;conclusion.$$

Conditions (pairs attribute = value) correspond to descriptors that are present in the premise part of the rule. Conclusion corresponds to the rule consequent part that present a class label. Rules presented in such a form can be considered as a compact form of knowledge representation. This form is simple and easily accessible from the point of view of understanding and interpreting knowledge represented by rules. Moreover, decision rules based on background knowledge can be employed in classification tasks, where a class label for a new object is assigned based on its conditions. Hence, decision rules can be applied in data mining tasks related to (i) knowledge representation and (ii) classification [27]. Taking into account these two perspectives, there are different measures used for rule evaluation and many different approaches for the induction of decision rules. The aim is to find patterns or regularities hidden in the data that are interesting and useful for users.

It should be noted that the minimization of length (number of conditions) and the maximization of support (which allows to discover major patterns in data) of decision rules are NP-hard problems [6,14]. The most part of approaches for construction of decision rules, with the exception of brute force, Boolean reasoning [28], and dynamic programming [6], cannot guarantee the construction of optimal rules, i.e., rules with minimum length or maximum support. Consequently, different heuristic approaches have been proposed in the literature [26,27,29,30]. Among them, greedy algorithms, genetic algorithms, ant colony optimization algorithms, approaches based on a sequential covering procedure, and many others can be mentioned.

#### 2.2.2. Decision Trees

Decision trees are often used as classifiers, as a means of knowledge representation, and as algorithms. A decision tree learning algorithm approximates a target concept using a tree representation, where each internal node corresponds to an attribute, and each terminal node known as a leaf corresponds to a class label. The root node is at the top and leafs are at the bottom of a tree.

Most of the algorithms for decision tree induction use a greedy approach and a top-down, recursive, divide-and-conquer technique. In general, the algorithm for decision tree induction starts with the tree, which initially contains a single root node that is associated with the objects included in a data set. Then, the instances are recursively partitioned into smaller subsets according to a given splitting criterion. It indicates the attribute chosen as the test condition and how the instances should be distributed to the child nodes of the constructed tree. The creation and expansion of a node is finished when the stop criterion is satisfied, for example, when all the instances associated with the node in the divided data set have the same class label. However, there are also other criteria that allow for the expansion of a node to be stopped earlier even if corresponding assigned instances have different decisions.

An advantage of decision trees is that by reading a tree from root to leaves, a decision (class label) is proposed for a considered case (object); it is also possible to see the reasons for choosing a given decision. This feature is a very important element used in the domain of applications aimed at supporting decision making. In addition, based on the decision tree, decision rules can be obtained.

There are many algorithms for decision tree induction. The most popular are [8,9,31,32]: CART (Classification and Regression Trees), ID3 (Iterative Dichotomiser 3), C4.5 (improved version of the ID3 algorithm, where “C” shows that algorithm was written in C and the 4.5 specifics version of this algorithm), Sprint (Scalable PaRallelizable INduction of decision Trees), Chaid (Chi-square automatic interaction detection), and their many modifications. There are also a variety of approaches based on meta-heuristics [33] such as genetic algorithms, simulated annealing, ant colony optimization, and many others. An important element during decision tree induction is selecting the best split, which allows for the partitioning of instances into two or more subsets that are associated with the nodes of the decision tree. Among the popular ones, measures based on entropy and the Gini index used in CART can be distinguished.

#### 2.2.3. Tests and Reducts

The construction of reducts and tests (super reducts) is closely connected with the feature selection area [34,35,36]. The aim of this domain is to select from the entire set of features only those attributes that are the most relevant while maintaining the descriptive and classification properties of the original feature space. Hence, this reduced set of attributes can be used instead of the entire attributes set for knowledge discovery. It is an important task, especially in areas where data sets contain a huge number of features, for example, in market basket analysis, stock trading, and sequence pattern discovery in bioinformatics.

Reduct is as an irreducible subset of features providing a satisfactory level of information about the considered target variable, which can be, for example, the accuracy of the classifier constructed based on the features contained in it. Therefore, from the classification point of view, a reduct can be interpreted as a minimal subset of attributes that has the same classification power as the entire set of features. Definitions for attribute reducts can be based on different criteria, for example, a reduct can also be considered as a minimal set of attributes that preserves the degree of dependency of the full set of attributes [37].

In the rough sets theory, where the construction of reducts constitutes one of the main research directions, decision super reduct (test) is defined as a subset of condition attributes that is sufficient for discerning any of the objects in a decision table with different class labels. A decision reduct is a test in the sense that each proper subset of this test is not a test for the considered problem.

Unfortunately, finding a reduct with minimum cardinality is an NP-hard problem. It is also known that the upper bound of a potential number of all reducts that can be found for a given dataset with *k* attributes is equal to k⌊k/2⌋. Taking into account that these issues represent high computational costs and complexity brought by the tasks of all reduct construction, different approaches and heuristics have been proposed for the construction of many reducts in some acceptable time. The popular ones are Boolean reasoning [28], genetic algorithms [38], greedy algorithms [39], fuzzy-rough approach, and others [14,40].

Based on the reduct constructed for a given decision table, decision rules can be induced from reduced sets of attributes. In this indirect method of rule induction, it is easy to see that the number of attributes which constitute a reduct is an important factor from the point of view of knowledge representation. Short reducts allow for the construction of short decision rules, which are more preferred from the point of view of understanding and interpretation by users.

#### 2.2.4. Association Rules

Association rule mining is one of the key and interesting methods of data mining and knowledge discovery. It aims to extract co-occurrences of items as well as associations and patterns hidden in the data. One of the most popular applications of association rules is the market basket analysis, which finds associations between different items that customers place in their shopping baskets. Other areas include business fields involving decision making and effective marketing, medical diagnosis, stock trading, and others.

There are different types of association rules, for example: boolean association rules, which are used in market basket analysis; qualitative association rules [11], which are induced from business data; spatial association rules [41]; multilevel association rules [42], and others [29]. In general, association rules are presented in the following form:

(2) X→Y,

where *X* and *Y* are sets of items.

Two main quality measures of association rules are support and confidence [15]. Rules that satisfy minimum thresholds of these measures indicated by a user are called strong association rules.

It should be also noted that there are many algorithms for construction of association rules, however the process of mining of association rules consists of two main stages: (i) find all frequent itemsets, i.e., they occur at least as frequently as a predetermined minimum support threshold, and (ii) generate strong association rules from the frequent itemsets, i.e., rules that satisfy minimum support and minimum confidence thresholds. The most popular algorithm based on mining frequent itemset is Apriori [43]. However, many other approaches were proposed by researchers, for example, algorithms that use frequent pattern growth approach [44], vertical data format [45], hash based technique, partitioning the data and others [46].

One very important task in data mining is the classification process. In this framework, association rules also have an application. The associative classification task aims to find association rules that have only the class label in the consequent part of the rule and which satisfies the minimum support and the confidence thresholds, the so-called Class Association Rules. There are many methods for the construction of classifiers, which differ in the approaches used for mining association rules and their selection [47].

## 3. Sets of Decision Tables

In this section, we deal with dispersed data represented as a finite set of decision tables with equal sets of attributes.

### 3.1. Main Notions

**Figure 1:**
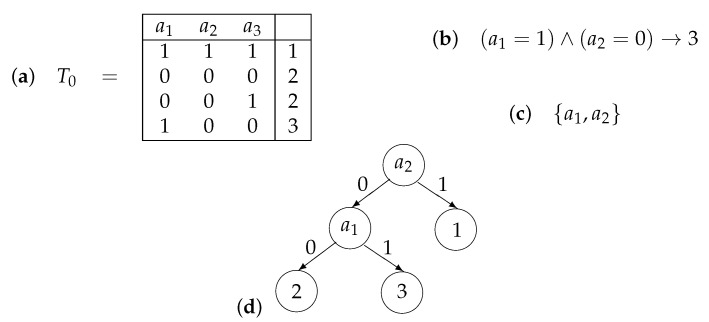
Considered objects: (**a**) decision table $T_{0}$, (**b**) decision rule for $T_{0}$ and row (1,0,0), (**c**) reduct for $T_{0}$, (**d**) decision tree for $T_{0}$.

A decision table *T* is a table filled with numbers from the set ω={0,1,2,…} of non-negative integers, in which columns are labeled with conditional attributes $a_{1},\ldots ,a_{n}$ and each row is labeled with a decision that is a number from ω (see Figure 1). We assume that equal rows in the table *T* are labeled with equal decisions, i.e., we consider only consistent decision tables. We associate the following problem with the table *T*: for a given row ρ of *T*, we should recognize the decision attached to ρ using values of the condition attributes from $\left\{a_{1},\ldots ,a_{n}\right\}$ in this row. To this end, we can use decision trees, rules, and test (reducts).

A decision tree Γ over *T* is a finite directed tree with a root, in which each internal node is labeled with an attribute from the set $\left\{a_{1},\ldots ,a_{n}\right\}$, edges leaving this node are labeled with pairwise different numbers from ω, and each leaf node is labeled with a decision from ω. For a given row $\rho =(\delta_{1},\ldots ,\delta_{n})$, the tree Γ work starts in the root of Γ. If the node under consideration is a leaf, then the number attached to this node is the result of the Γ work. Let the node under consideration be an internal node with an attribute $a_{i}$ attached to it. If there is an edge that leaves the considered node and is labeled with $\delta_{i}$, then we pass along this edge. Otherwise, the decision tree Γ finishes its work without a result. We say that Γ is a decision tree for *T* if, for any row of *T*, the work of Γ finishes in a leaf that is labeled with the same decision as the considered row (see Figure 1). We denote with Trees(T) the set of decision trees for *T*.

Any decision rule over *T* can be represented in the following form:

(3) $$\left(a_{i_{1}}=\sigma_{1}\right)\wedge \cdots \wedge \left(a_{i_{m}}=\sigma_{m}\right)\to t$$

where $a_{i_{1}},\ldots ,a_{i_{m}}\in \left\{a_{1},\ldots ,a_{n}\right\}$ and $\sigma_{1},\ldots ,\sigma_{m},t\in \omega$. This rule is called realizable for a row $\rho =\left(\delta_{1},\ldots ,\delta_{n}\right)\in \omega^{n}$ (it is possible that this row does not belong to *T*) if $\delta_{i_{1}}=\sigma_{1},\ldots ,\delta_{i_{m}}=\sigma_{m}$. This rule is called true for *T* if, for any row $\rho^{\prime }$ of *T*, such that rule (3) is realizable for $\rho^{\prime }$, the row $\rho^{\prime }$ is labeled with the decision *t*. We say that (3) is a rule for *T* and ρ if this rule is true for *T* and realizable for ρ (see Figure 1). We denote with Rules(T,ρ) the set of decision rules for *T* and ρ. One can show that (3) is a rule for *T* and ρ if (i) ρ is labeled with the decision *t* if ρ belongs to *T*, and (ii) if each row $\rho^{\prime }$ of *T*, which is labeled with a decision different from *t*, is different from ρ on at least one attribute from the set $\left\{a_{i_{1}},\ldots ,a_{i_{m}}\right\}$.

A test for *T* is a subset of the set of conditional attributes $\left\{a_{1},\ldots ,a_{n}\right\}$, such that any two rows from *T* with different decisions are different on at least one attribute from this subset. A reduct for *T* is a test for *T*, for which each proper subset is not a test (see Figure 1). We denote with Tests(T) the set of tests for *T*.

Let $T=\{T_{1},\ldots ,T_{k}\}$ be a finite nonempty set of decision tables, in which columns are labeled with the same conditional attributes $a_{1},\ldots ,a_{n}$. Each decision table from this set is consistent, but different tables from T can contain equal rows labeled with different decisions. Let ρ be a row of a decision table from T. We denote $Trees\left(T\right)={⋂}_{T_{i}\in T}Trees\left(T_{i}\right)$, $Rules\left(T,\rho \right)={⋂}_{T_{i}\in T}Rules\left(T_{i},\rho \right)$, and $Tests\left(T\right)={⋂}_{T_{i}\in T}Tests\left(T_{i}\right)$. In the next three sections, we will consider joint decision tables for these sets of common decision trees, rules, and tests (reducts) for T.

### 3.2. Joint Decision Tables for Decision Trees

Let $T=\{T_{1},\ldots ,T_{k}\}$ be a set of decision tables, in which the columns are labeled with the attributes $a_{1},\ldots ,a_{n}$. The set of decision tables T is called consistent if there are no two tables in T containing equal rows labeled with different decisions.

First, we show that if the set T is not consistent, then Trees(T)=∅. Since T is not consistent, there exist two tables $T_{i}$ and $T_{j}$ in T and a row ρ, such that ρ is a row of $T_{i}$ labeled with a decision *p*, ρ is a row of $T_{j}$ labeled with a decision *q*, and p≠q. Let us assume that Trees(T)≠∅ and Γ∈Trees(T). Then, the output of Γ for the row ρ should be equal to *p* and to *q* at the same time, but this is impossible. Therefore, Trees(T)=∅.

**Figure 2:**
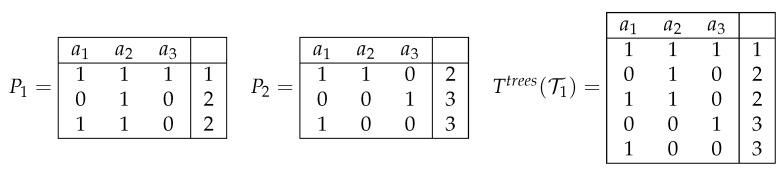
Joint decision table $T^{trees}\left(T_{1}\right)$ for the set of decision tables $T_{1}=\left\{P_{1},P_{2}\right\}$.

Let us assume now that the set T is consistent. With $T^{trees}\left(T\right)$, we denote a decision table in which columns are labeled with attributes $a_{1},\ldots ,a_{n}$, and the set of rows coincides with the union of sets of rows of the tables $T_{1},\ldots ,T_{k}$. Each row belonging to $T^{trees}\left(T\right)$ is labeled with the decision attached to this row in the tables from T which this row belongs to (see Figure 2). Note that the table $T^{trees}\left(T\right)$ can be constructed in polynomial time.

We now show that $Trees\left(T\right)=Trees(T^{trees}\left(T\right))$. Let Γ∈Trees(T). Then, for any $T_{i}\in T$ and any row ρ belonging to $T_{i}$, Γ returns the decision attached to ρ in $T_{i}$. Therefore, for any row ρ of $T^{trees}\left(T\right)$, Γ returns the decision attached to ρ, i.e., $\Gamma \in Trees(T^{trees}\left(T\right))$. Now, let $\Gamma \in Trees(T^{trees}\left(T\right))$. Then, for any row ρ of $T^{trees}\left(T\right)$, Γ returns the decision attached to ρ. Therefore, for any table $T_{i}\in T$ and any row ρ of $T_{i}$, Γ returns the decision attached to ρ in $T_{i}$, i.e., Γ∈Trees(T).

### 3.3. Joint Decision Tables for Decision Rules

Let $T=\{T_{1},\ldots ,T_{k}\}$ be a set of decision tables, in which columns are labeled with attributes $a_{1},\ldots ,a_{n}$. A row ρ of a decision table from the set T is called inconsistent if there are two tables in T that contain it and if the row ρ in these tables is labeled with different decisions. Otherwise, the row ρ is called consistent.

First, we show that if the row ρ is inconsistent, then Rules(T,ρ)=∅. Since ρ is inconsistent, there exist two tables $T_{i}$ and $T_{j}$ in T, such that ρ is a row of $T_{i}$ labeled with a decision *p*, ρ is a row of $T_{j}$ labeled with a decision *q*, and p≠q. Let us assume that Rules(T,ρ)≠∅. Then, the right-hand side of each rule from Rules(T,ρ) should be equal to *p* and to *q* at the same time, but this is impossible. Therefore, Rules(T,ρ)=∅.

**Figure 3:**
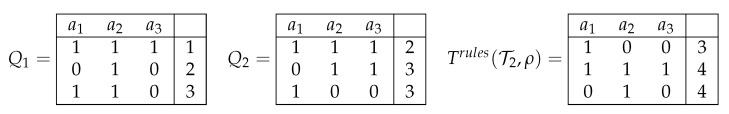
Joint decision table $T^{rules}\left(T_{2},\rho \right)$ for the set of decision tables $T_{2}=\left\{Q_{1},Q_{2}\right\}$ and row ρ=(1,0,0).

Let us assume now that the row ρ is consistent, and that it is labeled with the decision *t*. We denote with $T^{rules}\left(T,\rho \right)$ a decision table in which columns are labeled with attributes $a_{1},\ldots ,a_{n}$, the first row is ρ, and the set of all other rows coincides with the union of the sets of rows of the tables $T_{1},\ldots ,T_{k}$, which are labeled with decisions different from *t*. The first row of $T^{rules}\left(T,\rho \right)$ is labeled with the decision *t*, and all other rows are labeled with the decision t+1 (see Figure 3). We cannot keep the initial decisions for rows that are now labeled with t+1 since in this case, the table $T^{rules}\left(T,\rho \right)$ can be inconsistent. Note that the table $T^{rules}\left(T,\rho \right)$ can be constructed in polynomial time.

We now show that $Rules\left(T,\rho \right)=Rules(T^{rules}\left(T,\rho \right),\rho )$. Let ρ∈Rules(T,ρ) and ρ be equal to (3). Then, for any table $T_{i}$ from T, any row of $T_{i}$ labeled with a decision different from *t* is different from ρ on at least one attribute from the set $\left\{a_{i_{1}},\ldots ,a_{i_{m}}\right\}$. Therefore, any row of $T^{rules}\left(T,\rho \right)$ labeled with the decision t+1 is different from ρ on at least one attribute from the set $\left\{a_{i_{1}},\ldots ,a_{i_{m}}\right\}$, i.e., ρ∈ $Rules(T^{rules}\left(T,\rho \right),\rho )$. Now, let $\rho \in Rules(T^{rules}\left(T,\rho \right),\rho )$. Then, any row of $T^{rules}\left(T,\rho \right)$ labeled with the decision t+1 is different from ρ on at least one attribute from the set $\left\{a_{i_{1}},\ldots ,a_{i_{m}}\right\}$. Therefore, for any table $T_{i}$ from T, any row of $T_{i}$ labeled with a decision different from *t* is different from ρ on at least one attribute from the set $\left\{a_{i_{1}},\ldots ,a_{i_{m}}\right\}$, i.e., ρ∈Rules(T,ρ).

### 3.4. Joint Decision Tables for Tests (Reducts)

Let $T=\{T_{1},\ldots ,T_{k}\}$ be a set of decision tables, in which columns are labeled with attributes $a_{1},\ldots ,a_{n}$. Each decision table from this set is consistent, but different tables from T can contain equal rows labeled with different decisions. It is clear that for each table $T_{i}$ from T, the set of attributes $\left\{a_{1},\ldots ,a_{n}\right\}$ is a test. Therefore, Tests(T)≠∅.

**Figure 4:**
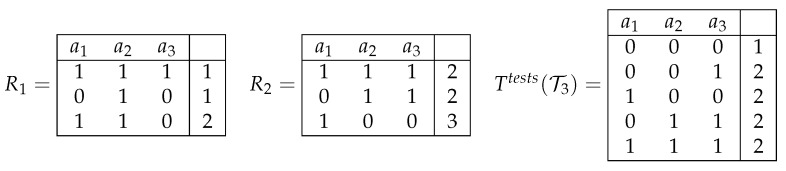
Joint decision table $T^{tests}\left(T_{3}\right)$ for the set of decision tables $T_{3}=\left\{R_{1},R_{2}\right\}$.

We denote with $T^{tests}\left(T\right)$ a decision table in which columns are labeled with attributes $a_{1},\ldots ,a_{n}$, the first row is filled with zeros, and the set of all other rows is constructed in the following way. For any table $T_{i}$ from T and any two rows $\rho_{1}$ and $\rho_{2}$ of $T_{i}$ labeled with different decisions, we add to the table $T^{tests}\left(T\right)$ the row $c(\rho_{1},\rho_{2})$ filled with numbers from the set {0,1}. For i=1,…,n, the row $c(\rho_{1},\rho_{2})$ has the number 1 in the *i*th position if and only if the rows $\rho_{1}$ and $\rho_{2}$ are different on the attribute $a_{i}$. The first row of the table $T^{tests}\left(T\right)$ is labeled with the decision 1. All other rows are labeled with the decision 2 (see Figure 4). It is clear that the rows $\rho_{1}$ and $\rho_{2}$ are different on an attribute $a_{j}$ if and only if the first row of the table $T^{tests}\left(T\right)$ and the row $c(\rho_{1},\rho_{2})$ are different on the attribute $a_{j}$. Note that the table $T^{tests}\left(T\right)$ can be constructed in polynomial time.

We now show that $Tests\left(T\right)=Tests(T^{tests}\left(T\right))$. Let B∈Tests(T). Then, for any table $T_{i}$ from T, any two rows from $T_{i}$ with different decisions are different on at least one attribute from *B*. Therefore, the first row of the table $T^{tests}\left(T\right)$ is different from all other rows of the table $T^{tests}\left(T\right)$ on the attributes from *B*, i.e., $B\in Tests(T^{tests}\left(T\right))$. Let $B\in Tests(T^{tests}\left(T\right))$. Then, the first row of the table $T^{tests}\left(T\right)$ is different from all other rows of the table $T^{tests}\left(T\right)$ on the attributes from *B*. Therefore, for any table $T_{i}$ from T, any two rows from $T_{i}$ with different decisions are different on at least one attribute from *B*, i.e., B∈Tests(T).

## 4. Sets of Information Systems

In this section, we deal with dispersed data represented as a finite set of information systems with equal sets of attributes.

### 4.1. Main Notions

An information system *I* is a table filled with numbers from the set ω={0,1,2,…} of non-negative integers, in which columns are labeled with attributes $a_{1},\ldots ,a_{n}$. Each row ρ of the information system *I* is interpreted as an object, and the number in the intersection of the row ρ and the column $a_{i}$ is interpreted as the value $a_{i}\left(\rho \right)$ of the attribute $a_{i}$ for the object ρ.

**Figure 5:**
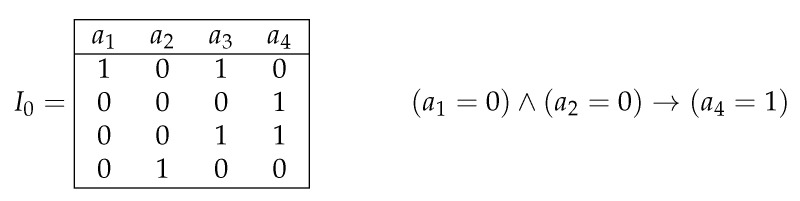
Information system $I_{0}$ and the association rule, which is based on the attribute $a_{4}$, true for the information system $I_{0}$, and realizable for the row (0,0,0,1).

Any association rule over the set of attributes $\left\{a_{1},\ldots ,a_{n}\right\}$ can be represented in the following form:

(4) $$\left(a_{i_{1}}=\sigma_{1}\right)\wedge \cdots \wedge \left(a_{i_{m}}=\sigma_{m}\right)\to \left(a_{j}=\sigma \right),$$

where $a_{j}\in \left\{a_{1},\ldots ,a_{n}\right\}$, $a_{i_{1}},\ldots ,a_{i_{m}}\in \left\{a_{1},\ldots ,a_{n}\right\}\setminus \left\{a_{j}\right\}$, and $\sigma_{1},\ldots ,\sigma_{m},\sigma \in \omega$. We will say that this rule is based on the attribute $a_{j}$. Rule (4) is called realizable for a row $\rho =\left(\delta_{1},\ldots ,\delta_{n}\right)\in \omega^{n}$ if $\delta_{i_{1}}=\sigma_{1},\ldots ,\delta_{i_{m}}=\sigma_{m}$. This rule is called true for the information system *I* if for any row $\rho^{\prime }$ of *I* such that rule (4) is realizable for $\rho^{\prime }$, $a_{j}\left(\rho^{\prime }\right)=\sigma$ (see Figure 5).

### 4.2. Joint Information Systems for Association Rules

Let $I=\{I_{1},\ldots ,I_{k}\}$ be a finite nonempty set of information systems, in which columns are labeled with the same attributes $a_{1},\ldots ,a_{n}$. Let $\rho =(\delta_{1},\ldots ,\delta_{n})$ be a row of an information system from I and $a_{j}\in \left\{a_{1},\ldots ,a_{n}\right\}$. We denote with $Arules(I,\rho ,a_{j})$ the set of association rules over the set of attributes $\left\{a_{1},\ldots ,a_{n}\right\}$, each of which is based on the attribute $a_{j}$, is realizable for the row ρ, and is true for each information system from I.

Our aim is to construct a so-called joint information system *J*, for which

(5) $$Arules\left(\left\{J\right\},\rho ,a_{j}\right)=Arules\left(I,\rho ,a_{j}\right).$$

**Figure 6:**
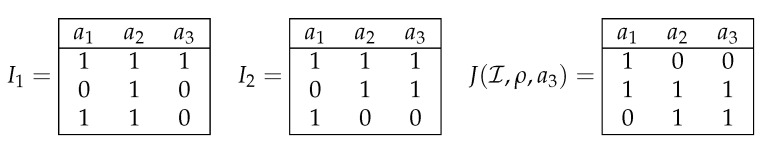
Joint information system $J(I,\rho ,a_{3})$ for the set of information systems $I=\{I_{1},I_{2}\}$, row ρ=(1,0,0), and attribute $a_{3}$.

In the information system $J=J(I,\rho ,a_{j}),$ columns are labeled with the attributes $a_{1},\ldots ,a_{n}$. This information system contains row ρ and all rows $\rho^{\prime }$ from the information systems $I_{1},\ldots ,I_{k}$, such that $a_{j}\left(\rho \right)\neq a_{j}\left(\rho^{\prime }\right)$ (we keep only one row from any group of equal rows) (see Figure 6). Note that the information system *J* can be constructed in polynomial time.

It is easy to show that the set of rules $Arules\left(\left\{J\right\},\rho ,a_{j}\right)\cup Arules\left(I,\rho ,a_{j}\right)$ is a subset of the set *A* of rules in the following form:

$$\left(a_{i_{1}}=\delta_{i_{1}}\right)\wedge \cdots \wedge \left(a_{i_{m}}=\delta_{i_{m}}\right)\to \left(a_{j}=\delta_{j}\right),$$

where $a_{i_{1}},\ldots ,a_{i_{m}}\in \left\{a_{1},\ldots ,a_{n}\right\}\setminus \left\{a_{j}\right\}$. To show that equality (5) holds, it is enough to prove that, for any rule r∈A, $r\notin Arules(\left\{J\right\},\rho ,a_{j})$ if and only if $r\notin Arules(I,\rho ,a_{j})$. It is clear that each rule from *A* is based on the attribute $a_{j}$ and is realizable for the row ρ. Let $r\notin Arules(\left\{J\right\},\rho ,a_{j})$. Then, the rule *r* is not true for *J*, and there exists a row $\rho^{\prime }$ from *J* such that *r* is realizable for $\rho^{\prime }$ and $a_{j}\left(\rho \right)\neq a_{j}\left(\rho^{\prime }\right)$. It is clear that $\rho^{\prime }$ is a row from an information system $I_{i}$ from I. Then, *r* is not true for $I_{i}$ and $r\notin Arules(I,\rho ,a_{j})$. Let $r\notin Arules(I,\rho ,a_{j})$. Then, there exists an information system $I_{i}\in I$ for which *r* is not true, and there exists a row $\rho^{\prime }$ from $I_{i}$ such that *r* is realizable for $\rho^{\prime }$ and $a_{j}\left(\rho \right)\neq a_{j}\left(\rho^{\prime }\right)$. It is clear that $\rho^{\prime }$ is a row from the information system *J*. Then, *r* is not true for *J*, and $r\notin Arules(\left\{J\right\},\rho ,a_{j})$. Thus, the equality (5) holds.

## 5. Conclusions

In this simple methodological paper, we have shown the problem of studying common decision trees for a dispersed set of decision tables with equal sets of attributes and how to reduce this to the study of decision trees for a single decision table. We accomplished the same for common decision rules and tests (reducts). The proposed approach allows us to generalize known methods in the study of single decision tables to the case of dispersed tables with equal sets of attributes.

We also showed the problem of studying common association rules for a dispersed set of information systems with equal sets of attributes and how to reduce this to the study of association rules for a single information system. The proposed approach allows us to generalize known methods in the study of association rules for single information systems to the case of dispersed information systems with equal sets of attributes.

The presented idea is different from the methods offered in the framework of distributed data mining or data warehouses. In our approach, the cost of communication in a distributed environment is limited to the construction of a joint tabular form. Then, depending on the aim of the data analysis, different existing algorithms for the induction of decision trees, rules, reducts, or association rules can be used. In the case of data warehouses, the main application is the use of OLAP tools for supporting business decisions. In the case of distributed data mining, collaboration among agents in the entire system and learning at the local level are important factors that are omitted in the proposed approach.

Future research will be connected with developing an algorithm for the induction of decision rules from distributed data. The proposed idea will be different from the one presented in this paper, since decision rules will be induced from a set of decision tables without the process of transforming the distributed data into a joint tabular form.

## Data Availability

Not applicable.
